# Enhancement of temozolomide stability by loading in chitosan-carboxylated polylactide-based nanoparticles

**DOI:** 10.1007/s11051-017-3756-3

**Published:** 2017-02-16

**Authors:** Antonio Di Martino, Pavel Kucharczyk, Zdenka Capakova, Petr Humpolicek, Vladimir Sedlarik

**Affiliations:** Centre of Polymer Systems, University Institute, Tomas Bata University in Zlín, tr. T. Bati 5678, 76001 Zlin, Czech Republic

**Keywords:** Temozolomide, Nanoparticles, Drug delivery, Chitosan, Polylactic acid, Encapsulation, Nanomedicine

## Abstract

In the presented work, amphiphilic nanoparticles based on chitosan and carboxy-enriched polylactic acid have been prepared to improve the stability of the pro-drug temozolomide in physiological media by encapsulation. The carrier, with a diameter in the range of 150–180 nm, was able to accommodate up to 800 μg of temozolomide per mg of polymer. The obtained formulation showed good stability in physiological condition and preparation media up to 1 month. Temozolomide loaded inside the carrier exhibited greater stability than the free drug, in particular in simulated physiological solution at pH 7.4 where the hydrolysis in the inactive metabolite was clearly delayed. CS-SPLA nanoparticles demonstrated a pH-dependent TMZ release kinetics with the opportunity to increase or decrease the rate. Mass spectroscopy, UV-Vis analysis, and in vitro cell tests confirmed the improvement in temozolomide stability and effectiveness when loaded into the polymeric carrier, in comparison with the free drug.

**Electronic supplementary material:**

The online version of this article (doi:10.1007/s11051-017-3756-3) contains supplementary material, which is available to authorized users.

## Introduction

Temozolomide (TMZ) (8-carbamoyl-3-methylimidazo[5,1-d]-1,2,3,5-tetrazin-4-(3H)-one) belongs to the class of alkylating agents known as imidazotetrazines and represents one of the major chemotherapeutic agents used in glioblastoma multiforme (GBM) treatment (Appel et al. 2012; Dresemann 2010; Hegi et al. 2005). It is a prodrug, which crosses the blood–brain barrier, and is converted via hydrolysis under physiological conditions in the active form 5-(3-methyl-triazen-1-yl) imidazole-4-carboxamide (MTIC). TMZ degradations in the MTIC occur after uptake by GBM cells in the brain. The MTIC methylates DNA in the O^6^ position of guanine residues, causing cytotoxic DNA damage (Meer et al. 1986).

TMZ hydrolysis can also take place in the blood stream. Nevertheless, the MTIC is not able to cross the blood–brain barrier and exhibits low cell absorption, thereby diminishing therapeutic efficiency.

According to the literature (Appel et al. 2012; Lagona et al. 2005), improving the half-life of TMZ under physiological conditions would promote greater accumulation of the TMZ accessing the GBM site, prior to degradation. Consequently, the drug would be more effective, and lower doses could be utilized to maintain the current therapeutic window.

Different approaches have been reported, ranging from chemical modification to coating procedures, although recent years have witnessed considerable interest in techniques for TMZ encapsulation into polymeric microparticles and nanoparticles.

Nanoparticles, thanks to their unique properties, are under active study as drug delivery carriers for the treatment of disease (Koo et al. 2006; Fahr and Liu 2007). Polymeric-based nanoparticles have become tremendously important in developing such systems due to their tunable characteristics and potential to entrap, dissolve, or attack a wide range of drugs or other active pharmaceutical ingredients (APIs) (McCarron and Hall 2004; Lu et al. 2006; Wen and Kesari 2008; Jeyarama et al. 2016; Agarwala and Kirkwood 2000a, b).

Particular interest has been shown in biopolymers, especially polysaccharides, due to peculiarities such as a large number of reactive groups, a wide range of molecular weights (M_W_), and variation in chemical composition according to the source (Sinha and Kumria 2001), these properties allowing for preparation of a carrier with well-defined properties.

Chitosan belongs to the group of polysaccharides and can easily be prepared through partial or total deacetylation of chitin. It is a copolymer of *d*-glucosamine and *N*-acetyl-*d*-glucosamine, linked by *β*-(1,4) glycosidic bonds. It has been widely applied in the pharmaceutical and medical sectors as a consequence of the favorable biological properties pertaining to it, such as biodegradability (Bowman and Leong 2006). Chemically modifying CS, in particular through grafting procedures, appears promising for developing highly tailored drug carriers for controlled and sustained delivery of selected APIs. As reported in our paper and other published works, modification of CS by introduction of polylactic acid chains presented several advantages (Wang et al. 2014a, b; Ge et al. 2015; Huang et al. 2013; Di Martino and Sedlarik 2014).

Several works have been published regarding the encapsulation of TMZ in nanocarrier of various natures. However, the main concern was to improve the selectivity of the drug, avoid the development of resistance mechanism from the cancer cells, and reduce side effects related to the administration frequency (Jain et al. 2014; Xu et al. 2015). Only a few works, dealing with the use of nanoparticles, in particular those based on polymers, improving TMZ stability in physiological conditions by delaying the hydrolysis process, are reported.

Most of the studies focused on the increment of stability deal with the chemical modification of TMZ. It represents a valid strategy, which already demonstrated good results, as a lot has been published about imidazotetrazines chemistry (Matosiuk et al. 2002; El-Sharief et al. 2006; Tyndall et al. 2015). However, the chemical modification is a longer and more complicated procedure than encapsulation in specific nano-sized carrier, which is—in some cases—fast, highly reproducible, and solvent free.

The presented work is focused on the preparation of amphiphilic nanoparticles based on CS grafted by carboxy-enriched polylactic acid (SPLA), with the aim of loading and improving the stability of TMZ in simulated physiological conditions, in particular at pH >7. Nanoparticles based on CS-grafted SPLA (CS-SPLA) were characterized in terms of dimension, surface properties, and stability in different media. The capacity of the prepared nanoparticles to load, release for an extended period, and improve TMZ stability by delaying hydrolysis, in particular at pH >7, were demonstrated by UV–Vis spectrophotometry and LC-MS analysis. Cytotoxicity tests on two mouse fibroblast cell lines (MEF and NHT/3T3) demonstrated the safety of the bare CS-SPLA nanoparticles and their capability to enhance TMZ stability and efficacy, compared to the free drug.

## Materials and methods

### Materials

Chitosan (20–300 cP, 1 wt.% in 1% acetic acid (25 °C, Brookfield), D.D 75–85%), dextran sulfate (M_w_ 40 kDa), dimethyl sulfoxide in deuterated form (DMSO-d6), pentetic acid (PA) (*N,N*-Bis(2-bis(carboxymethyl)amino]ethyl)glycine, ≥99.5% ), methanesulfonic acid (MSA, ≥95%), hydrochloric acid (HCl, 30% for trace analysis), doxorubicin hydrochloride, and 5-fluorouracil were supplied by Sigma Aldrich. L-Lactic acid (80% water solution) was purchased from Merci s.r.o., Czech Republic. Sodium hydroxide, sodium phosphate, potassium phosphate, and potassium hydroxide were bought from IPL Lukes, Uhresky Brod, Czech Republic. Acetic acid CH_3_CO_2_H (HPLC grade) was purchased from Chromspec, Brno, Czech Republic. The solvents acetone and methanol, the indicator phenolphthalein, and potassium hydroxide (all analytical grade) were bought from IPL Lukes, Uhersky Brod, Czech Republic. Tetrahydrofuran (HPLC grade) was purchased from Chromservis, Czech Republic.

### Methods

#### Synthesis and characterization of SPLA and CS-SPLA conjugate

SPLA was synthetized and characterized following a procedure reported elsewhere (Kucharczyk et al. 2016). In brief, 1 g of pentetic acid (PA) was mixed with lactic acid water solution and heated at 110 °C for 1 h under reflux. Then, 0.5 wt.% of methanesulfonic acid was added and the temperature raised to 130 °C. Afterward, pressure was decreased in two stages to <1 kPa. The resulting product was allowed to cool down at room temperature, after which it was dissolved in acetone. The polymer solution was precipitated into a mixture of chilled methanol and distilled water 1:10 (*v*/v), then filtrated, washed with water and methanol, and dried at 30 °C for 48 h in a vacuum oven (10 kPa).

The concentration of –COOH groups in SPLA was determined by titration in dichloromethane with 0.01 M KOH ethanol solution using phenolphthalein as an indicator. The concentration of –COOH was obtained according to the following equation:

1 $$ {C}_{C OOH}=\left(\frac{\left( a- b\right)\ast N}{m}\right) $$

where *a* and *b* are the volumes (mL) for titration of the sample and blank, respectively; *N* the normality of KOH (mol/L); and *m* the sample weight (g).

The molecular weight of the SPLA obtained was characterized by GPC (Agilent HT-GPC 220) equipped with a dual detection system (refractive index and viscometric detector), PL gel-mixed bed columns (1× Mixed-B, 300 × 7.8 mm, 10 μm particles +1× Mixed-D, 300 × 7.8 mm, 5 μm particles +1× Mixed-E, 300 × 7.8 mm, 3 μm particles) at 40 °C in THF. The flow rate was set at 1.0 mL min^−1^ and the injection volume at 100 μL. The GPC system was calibrated with narrow polystyrene standards ranging from 162 to 72,000 g mol^−1^ (Polymer Laboratories Ltd., UK). The weight average molar mass M_w_, number average molar mass M_n_, and molar-mass dispersity (Đ = M_w_/M_n_) were determined from the peak corresponding to the polymer.

The amphiphilic polymer CS-SPLA was synthetized in accordance with a procedure reported previously (Di Martino and Sedlarik 2014), based on a coupling reaction between CS amino groups and PLA carboxylic groups. CS (0.5 g) was dissolved in an aqueous solution of 1% *v*/*v* acetic acid at 1 mg/mL concentration, while 0.5 g of SPLA, EDC, and *N*-hydroxysuccinimide (NHS) (at the molar ratio SPLA/EDC/NHS = 1:1.5:3) was dissolved in 50 mL of chloroform. Afterward, the solution containing SPLA was added to CS and kept under vigorous stirring for 48 h at room temperature. The reaction was stopped and the final product was precipitated by adding NaOH 0.1 M, then centrifuged for 15 min at 14,000 rpm, accurately washed with water and freeze-dried.

The physicochemical structure of the prepared polymer (SPLA) was analyzed by ^1^H-NMR (Varian Unity Inova at 400 MHz). The occurrence of the coupling reaction was confirmed by Fourier Transform infrared spectroscopy–attenuated total reflectance (FTIR-ATR) analysis (on a Nicolet iS5 FTIR Spectrometer equipped with an iD5 ATR accessory and ZnSe crystal, at resolution 4 cm^−1^, with 64 scans).

### Preparation and characterization of CS-SPLA nanoparticles loaded with TMZ

The nanoparticles were obtained by dissolving CS-SPLA (2 mg/mL) in acetic acid solution (pH 5.5) and DS in distilled water (0.5 mg/mL). A water solution containing TMZ (0.5 mg/mL) was prepared, added to the DS solution, and kept under stirring for 30 min. In order to avoid TMZ hydrolysis, the solution was acidified to pH 6 by acetic acid. Afterward, the solution containing DS and TMZ was added dropwise to the CS-SPLA solution and kept under vigorous stirring for 1 h at 40 °C. The pH of the solution was maintained at pH 5.5 for the entire process.

An aliquot of the solution containing the nanoparticles was withdrawn and filtered (0.45 μm) to remove the presence of dust and aggregates and analyzed by photon correlation spectroscopy (PCS), so as to determine the average diameter of the nanoparticles, as well as by ζ-potential (Nano ZS Malvern Instruments, UK). The remainder was centrifuged at 14,000 rpm for 15 min, and the pellet recovered and freeze-dried.

The shape of the TMZ loaded and unloaded freeze-dried CS-SPLA nanoparticles was investigated by scanning electron microscopy (PHENOM desk top SEM).

The stability of the nanoparticles in solution constitutes an important feature for evaluation. The change in dimension and ζ-potential of bare nanoparticles over time was investigated by PCS in two different environments—preparation media (pH 5.5) and phosphate buffer (pH 7.4) at room temperature.

TMZ encapsulation efficiency (EE) in the CS-SPLA nanoparticles was determined by UV–Vis spectrophotometry (Cary 300 Varian) at 327 nm. The concentration of the drugs was obtained from the calibration curve of the free drug in solution. EE values were obtained by the following equation:

2 $$ \mathrm{EE}\;\left(\%\right)=\left(\frac{D_t-{D}_f}{D_t}\right)\times 100 $$

where *D*_*t*_ represents the total amount of drug loaded (mg) and *D*_*f*_ the amount of free drug detected in the supernatant (mg).

The swelling behavior of the obtained material was determined following the reported procedure (Bajpai and Anjali 2003).

Nanoparticles (0.5 g) were allowed to swell in a defined volume (50 mL) of media and taken out afterward; then, the superficial water was removed and weighed. The weight of the swollen nanoparticles was monitored at intervals of 2 min till no gain in weight was recorded, indicating that equilibrium had been reached.

The following equation was used to determine the percentage of swelling (*S*):

3 $$ S=\frac{W_s-{W}_d}{W_d}\times 100 $$

where *W*_s_ is the weight (mg) of the nanoparticles in the state of swelling, while *W*_d_ is weight (mg) in lyophilized form (powder) (Bajpai et al. 2012).

#### TMZ release and stability studies

In vitro release studies were carried out in phosphate buffer solution (*PBS* pH 7.4) and simulated gastric fluid (SGF pH 2) at 37 °C.

In brief, 50 mg of loaded nanoparticles were suspended in 50 mL of media, following which they were kept at 37 °C and orbitally shaken at 120 rpm (on a Stuart Orbital GFL 3033 Shaking Incubator). At predetermined time intervals, an aliquot (3 mL) was withdrawn and analyzed on a UV–Vis spectrophotometer. The dissolution medium was replaced with a fresh one to maintain total volume. The amount of drug released (*DR*) was determined by the following equation:

4 $$ \mathrm{DR}\left(\%\right)=\left(\frac{D_t}{D_0}\right)\times 100{} $$

where *D*_*t*_ (mg) represents the amount of drug released at time *t*, and *D*_*0*_ (mg) is the amount of drug loaded. All studies were conducted in triplicate.

Concentration (*C*) data were evaluated by applying a zero-order (Eq. 5) and first-order equation (Eq. 6) with GraphPad Prism software (Version 6.04, San Diego, CA, USA):

5 $$ {Q}_t={Q}_0+ kt $$

where *Q*_*0*_ represents the initial amount of drug (mg), *Q*_*t*_ the cumulative amount of drug (mg) released at time *t* (h), and *k* (h^−1^) the release constant;

6 $$ C={C}_{\max}\times \left(1-{e}^{kt}\right) $$

*C* is the cumulative concentration (mg drug/mg polymer) of the drug released at given time *t* (h), *C*_max_ represents the maximum value of concentration that can be released from the tested system under the given conditions (mg drug/mg polymer), and *k* is the kinetic constant (h^−1^) that represents the intensity of release from the particles at the initial time (*t*).

In order to prove that no alteration in TMZ structure occurred after encapsulation and release, analysis in the form of LC-MS (6530 Accurate-Mass Q-TOF LC/MS Agilent Technologies, ion mode positive, ionization ESI, collision energy 10 eV) was carried out on solutions presenting TMZ release after 6 and 24 h.

TMZ (10 μg/mL) dissolved as the free drug in the tested media was used as the control. Chromatographic separation was achieved using a column (ZORBAX Extended-C18, 2.1 × 50 mm, 1.8-Micron, 600 Bar, Agilent) and mobile phase with 60% water (containing 0.1% of formic acid) and 40% methanol at the flow rate of 0.3 mL/min. The sample compartment was maintained at 37 °C, while the column stood at 30 °C ± 1 °C, and the wavelength was monitored at 325 nm; injection volume equaled 2 μL.

#### Cytotoxicity and cell morphology

Cytotoxicity testing was performed using a mouse embryonic fibroblast continuous (ATCC CRL-1658™ NIH/3T3) cell line. The culture medium comprised ATCC–formulated Dulbecco’s modified Eagle’s medium (BioSera, France), containing 10% calf serum (BioSera, France) and penicillin/streptomycin at 100 U mL^−1^ (PAA Laboratories GmbH, Austria).

The tested samples were diluted to concentrations of 5 μg mL^−1^ in the culture medium. Cytotoxicity testing was conducted according to the EN ISO 10993-5 standard, with modification. Cells were precultivated for 24 h, and the culture medium was subsequently replaced with dilutions of samples. As a reference, cultivation in pure medium without the presence of samples was used. In order to assess cytotoxic effect, an MTT assay (Invitrogen Corporation, USA) was performed after 24, 48, and 72 h of cell cultivation with the presence of samples. All the tests were performed in quadruplicate. Absorbance was measured at 570 nm by an Infinite M200 Pro NanoQuant absorbance reader (Tecan, Switzerland). Dixon’s Q test was applied to remove outlying values and mean values were calculated. Cell viability is shown as the percentage of cells present in each respective extract relative to the cells cultivated in pure extraction medium without colloidal polyaniline (100% viability).

Cells were precultivated for 24 h, and the culture medium was subsequently replaced with dilutions of samples. After 24 h of exposure, staining with Hoechst 33258 (Invitrogen, USA) and ActinRed™ 555 (Thermo Fisher Scientific, USA) was utilized to determine cell morphology. Prior to this the cells had been fixed and permeabilized. The cells were fixed using 4% formaldehyde (Penta, Czech Republic) for 15 min, washed by PBS, and subsequently poured with 0.5% Triton X-100 (Sigma-Aldrich, USA) for 5 min to facilitate permeabilization. Following this, the cells were washed three times by PBS (Invitrogen, USA). The required amount of PBS—two drops per 1 mL of ActinRed™ 555 and 5 μg mL^−1^ of Hoechst 33258—was added and left to incubate for 30 min in the dark. The changes in cell morphology were observed on an Olympus inverted fluorescent microscope (Olympus, IX 81).

### Statistical analysis

Data obtained via the DLS and UV–Vis experiments underwent one-way analysis of variance (ANOVA) with a Tukey’s post hoc multiple comparison test using GraphPad Prism software (Version 6.04, San Diego, CA, USA). Probability values (*p*) of less than 0.05 were considered statistically significant.

## Results and discussions

### SPLA and CS-SPLA conjugate characterization

According to the GPC data, SPLA exhibits slightly higher M_n_ (3.8 kg/mol) and M_w_ (5.5 kg/mol) values than the reference linear PLA, which showed 2.4 and 4.1 kg/mol, respectively. This indicates that several lactic acid monomers were attached to the PA carboxylic groups, producing fractions with higher molecular weights.

Proof of the branched structure of SPLA stems from intrinsic viscosity values (IVs). In fact, SPLA possesses an IV of 0.045 dL/g, which is lower than linear PLA (0.07 dL/g), despite presenting a higher molecular weight (Kucharczyk et al. 2016).

**Fig. 1:**
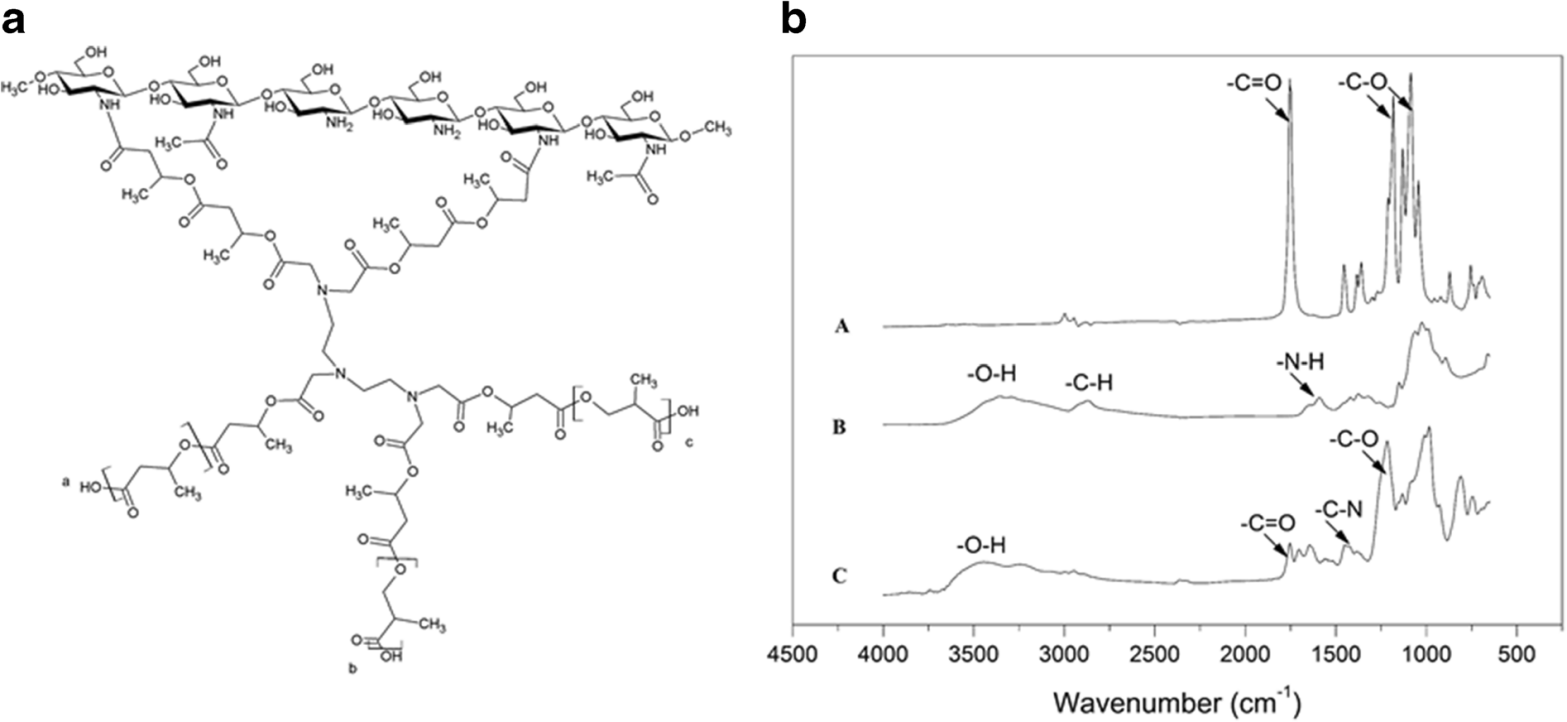
**a** Chemical structure of CS-SPLA (*a*, *b*, and *c* represent other possible points for the CS link). **b** FTIR-ATR spectra related to SPLA (*track A*), CS (*track B*) and the CS-SPLA product obtained (*track C*)

The most significant information derived from ^1^H-NMR spectroscopy relates to the COOH/OH ratio, indicating a significant deviation in SPLA (COOH/OH ratio = 3.45) compared to linear PLA (COOH/OH ratio close to 1). It clearly shows that a certain amount of the lactic acid OH group had successfully reacted with the COOH groups of pentetic acid (PA), thereby creating a branched structure (Fig. 1a). ^1^H-NMR spectra are reported in supportive information section.

Figure 1b presents FTIR-ATR spectra related to CS, SPLA, and the CS-SPLA product.

According to the FTIR-ATR data reported (Wang et al. 2014a, b), the more representative peaks for CS are at 3313 cm^−1^ (–O–H stretching), 2873 cm^−1^ (–C–H), 1585 cm^−1^ (NH_2_ deformation), and 1045 cm^−1^ (C–O–C). SPLA shows a typical signal for PLA (Drumright et al. 2000); these are at 2950 cm^−1^ (–CH– stretching); 1750 cm^−1^ (C=O stretching); 1452 cm^−1^ (CH_3_ bending); 1381 cm^−1^ and 1361 cm^−1^ (–CH– deformations and asymmetric bending); 1267 cm^−1^ (C–O stretching ); 1183 cm^−1^, 1128 cm^−1^, and 1084 cm^−1^ (C–O–C stretching); and finally 1043 cm^−1^ (−C-CH_3_ bending). The CS-SPLA spectra presents peaks at 1747 cm^−1^ (C=O stretching), 1648 cm^−1^—related to the amide bond between CS amino groups and SPLA carboxylic groups, and 1407 cm^−1^, representing the C–N stretching that proves the occurrence of a coupling reaction.

**Fig. 2:**
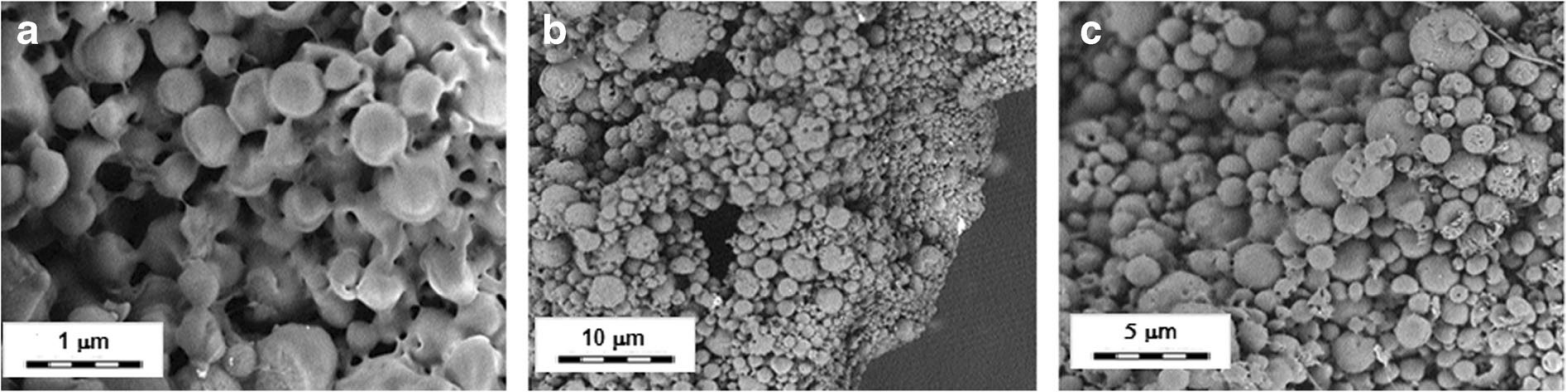
SEM micrographs of **a** unloaded and **b**, **c** loaded CS-SPLA freeze-dried nanoparticles. *Scale bars* **a** 1 μm, **b** 10 μm, and **c** 5 μm

The SEM micrographs (Fig. 2a–c) reveal that the nanoparticles are spherical in shape but possess a greater diameter than values recorded by DLS. As reported in the published works (Pikal 2002; Abdelwahed et al. 2006a, b, c), the increase in dimension can be attributed to the freeze-drying treatment performed without the use of cryo or lyo protectors. The freezing and drying processes generate stress that destabilizes the nanoparticle suspension. During the freezing stage, a phase separation into ice and cryo-concentrated solution occurs. In the suspension of nanoparticles, the cryo-concentrated phase is composed of nanoparticles and other components such as buffers and unloaded drugs (Abdelwahed et al. 2006a, b, c). This high concentration of the particulate system may induce aggregation and in some cases irreversible fusion of the nanoparticles. Moreover, ice crystallization places a mechanical stress on the nanoparticles, leading to destabilization of the same (Abdelwahed et al. 2006a, b, c).

However, as reported in the inner pane of Fig. 2b, the distribution of diameters of the freeze-dried nanoparticles after dissolution in preparation media and ultrasound treatment indicates that the aggregates are not fully irreversible.

### Nanoparticle characterization and TMZ loading

CS-SPLA complexes with DS forming nanoparticles made of hydrophobic (SPLA) and hydrophilic (CS) domain, in mildly acidic aqueous solution (pH 5.5). The dimension, stability over time, and ζ-potential of the CS-SPLA nanoparticles (loaded and unloaded) dispersed in said preparation media (pH 5.5) were investigated by PCS and a ζ-potential analyzer. The results are given in Table 1.

**Table 1:** Average dimension and ζ-potential of unloaded and loaded CS-SPLA nanoparticles and quantification of TMZ loading capacity

Average diameter (nm)		ζ-pot. (mV)		Encapsulation efficiency (%)	Loading capacity	mg TMZ/mg carrier
Unloaded	Loaded	Unloaded	Loaded			
165 ± 13	171 ± 15	+31 ± 2	+32 ± 3	81 ± 3	0.15 ± 0.01	0.26 ± 0.03

Table 1 presents results for nanoparticles loaded and unloaded with TMZ.

PCS reveals the average diameter of unloaded nanoparticles lies in the range 150–180 nm in the preparation media at room temperature.

**Fig. 3:**
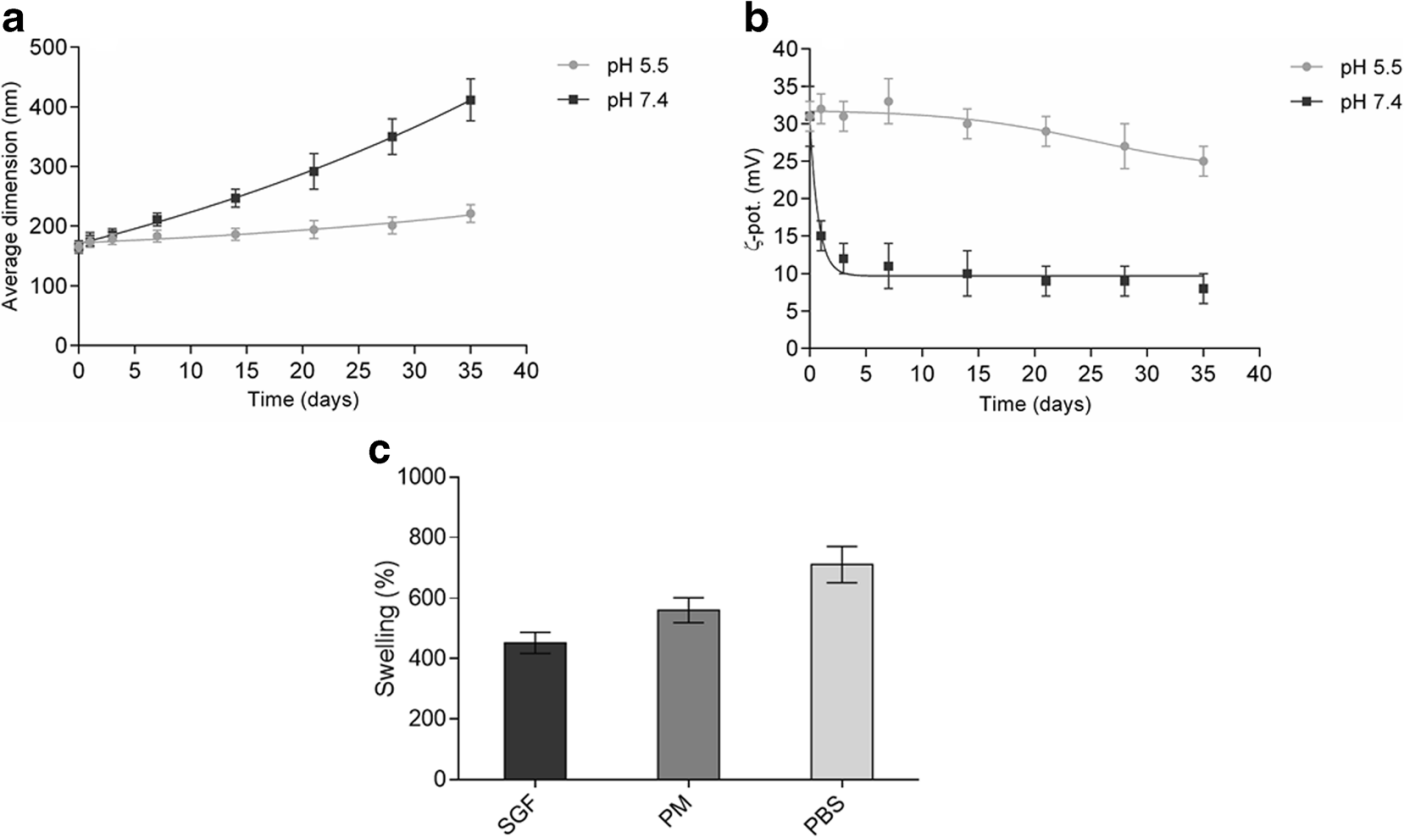
Evaluation of nanoparticle stability in terms of **a** average diameter and **b** ζ-potential variation over time in preparation media (pH 5.5) and physiological solution (pH 7.4) at room temperature. **c** Swelling percentages for CS-SPLA in different media. Simulated gastric fluid (SGF, pH 2), preparation media (PM, pH 5.5), and PBS (phosphate buffer, pH 7.4)

Drug loading does not influence the *ζ*-potential, which is around 30 mV. Despite the fact that the value falls in the interval which is linked to incipient instability/moderate stability, the prepared system showed good stability in physiological conditions (pH 7.4) and preparation media (pH 5.5) (Fig. 3).

Evaluating the stability of polymeric nanoparticles in biological-like environments is critical to devising optimal preparations and to developing notions on the fate of nanoparticles after administration (Lazzari et al. 2012). Herein, the stability of the nanoparticles in the preparation media (pH 5.5) and phosphate buffer (pH 7.4) was researched in terms of change in average diameter and *ζ*-potential over time.

The results obtained demonstrated the reasonable stability of the nanoparticles under the given preparation conditions (pH 5.5), showing an increase in size by 12% after 2 weeks and 35% after 1 month. These results are in agreement with those from the literature (You et al. 2013; Kumari et al. 2010) and confirm the stability of CS-SPLA nanoparticles in solutions. However, shifting the pH of the media from 5.5 to 7.4 led to remarkable decreases in stability. At pH 7.4, the nanoparticles were stable for up to 5 days, growing in dimension by around 14%. Afterward, aggregation and precipitation phenomena began causing a rise in size by up to 150% compared to that at the outset. Ultrasound treatment destroys the aggregates formed, restoring dimensions to values comparable with those initially. However, after 1–2 days, the aggregates reformed.

The values obtained fit with other data previously published on polymeric nanoparticles, which were mostly based on polybutyl cyanoacrylate (PBCA) (Tian et al. 2011), poly (methyl malate) (Lanz-Landázuri et al. 2011), or PLGA (Zhang and Gao 2007) for TMZ encapsulation (Cho et al. 2008; Ling et al. 2012).

In the prepared system, CS-SPLA, at pH 5.5, and all the functional groups—amino and carboxy—were in an ionic state, allowing strong intra-electrostatic and inter-electrostatic interactions between the chains. Moreover, the ζ-potential value of +30 mV evidences a certain stability that is due to repulsive forces between the nanoparticles. The amino groups became less protonated concurrently with increase in pH, which favored aggregation. It has to be stated that an important role is played by the concentration of the nanoparticles. The results presented relate to the concentration of 1 mg/mL, which could also substantiate the long-term stability recorded. A remarkable increase in the viscosity of the solution occurred when particle concentration equaled 10 mg/mL. Under the circumstances, not only did the number of nanoparticles in the considered volume increase but also mobility decreased, thereby favoring the formation of aggregates even in the preparation media.

Table 1 and Fig. 3b resume the ζ-potential of bare and loaded nanoparticles and the variation over time. No substantial effect is observed over time in the preparation media (pH 5.5). Conversely, at pH 7.4, two phases are identified. The first of these is where a sharp drop off in ζ-potential occurs, followed by the other, where ζ-potential values are stable over time. The rapid decrease in ζ-potential is triggered by the rise in pH levels. Actually, solid nanoparticles were dissolved in the media at pH 5.5, and afterward, the pH was increased to 7.4 by adding phosphate buffer (pH 7.4).

The TMZ was loaded into the CS-SPLA nanoparticles at a stage during preparation in order to obtain higher EE values (Table 1).

The TMZ EE for CS-SPLA stands at approximately 80%, with a loading capacity of 0.15 in the preparation media (pH 5.5). According to the reported data, the amount of TMZ (mg) per mg of carrier is around 0.25; this is comparable to—or in some cases higher than similar polymeric systems reported in the literature (Zhu and Liao 2015). Examples include TMZ loaded into poly (d,l lactide-co-glycolide), where entrapment efficiency ranged at 60–80% depending on the drug to polymer ratio, and poly butyl cyanoacrylate (PBCA), which has also been used as a vehicle for TMZ (Tian et al. 2011), exhibiting an entrapment efficiency of up to 45%. Additionally, poly methyl-malate nanoparticles have been prepared and loaded with TMZ in the past, demonstrating merely 20–40% of entrapment efficiency (Lanz-Landázuri et al. 2011).

Modifying the CS backbone by introducing a hydrophobic SPLA side chain directly influences the formation process of the nanoparticles as well as accommodation of the drug inside the structure (Juillerat-Jeanneret 2008).

In the encapsulation of various anticancer drugs (Tian et al. 2011; Allard et al. 2009), it has been demonstrated that the presence of hydrogen bonds between the drugs and CS plays a key role in the rate of loading; in the system under consideration, the OH and NH groups constitute donors while the acceptors comprise O and N.

According to data available on comparable systems (Tian et al. 2011), in the case of CS-SPLA, the drug tends to stay away from the CS backbone and situate itself closer to any hydrophobic chains, causing a deficiency in electrostatic interactions, which is in contrast to behavior with hydrophobic types.

Conversely, in unmodified CS, the drugs are expected to position themselves closer to the CS backbone, thereby causing more intense electrostatic interactions (Tian et al. 2011). The high encapsulation efficiency obtained by CS-SPLA could be ascribed to its greater capacity for hydrophobic interaction in holding the drug inside the system, as compared to electrostatic interaction (Juillerat-Jeanneret 2008; Baker et al. 1999).

Swelling behavior in different media represents a significant parameter for evaluation, as it directly influences release kinetics, in particular when the driving force is diffusion. The swelling percentages for CS-SPLA in media at various pH levels are illustrated in Fig. 3. The presence of SPLA has a certain impact on the swelling of CS-SPLA. The swelling behavior of unmodified CS and CS grafted with linear PLA-based nanoparticles was reported in a previous work by the authors (Di Martino et al. 2015, Di Martino and Sedlarik 2014). The results obtained demonstrated that the presence of SPLA influenced at different intensities the displacement of media molecules through the polymeric chains of the system. As the pH rises from 2 in SGF to 7.4 in PBS, there is a concurrent increase in the percentage of swelling. This is explained by the pK_a_ values for the NH_2_, COOH, and SO_3_ groups involved in forming the nanocomplexes. Raising the pH led to weakened electrostatic interactions between the NH_3_^+^ and COO^−^ groups, due to deprotonation of NH_3_^+^ to NH_2_. This permits faster diffusion of the media inside the system (Pasparakis and Bouropoulos 2006; Gasmi et al. 2015). The permeability of the media can also be converted in response to an alteration in environmental pH, which represents a desirable characteristic for a system with pH-sensitive controlled release and controllable swelling ability.

### TMZ release kinetics and stability studies

**Fig. 4:**
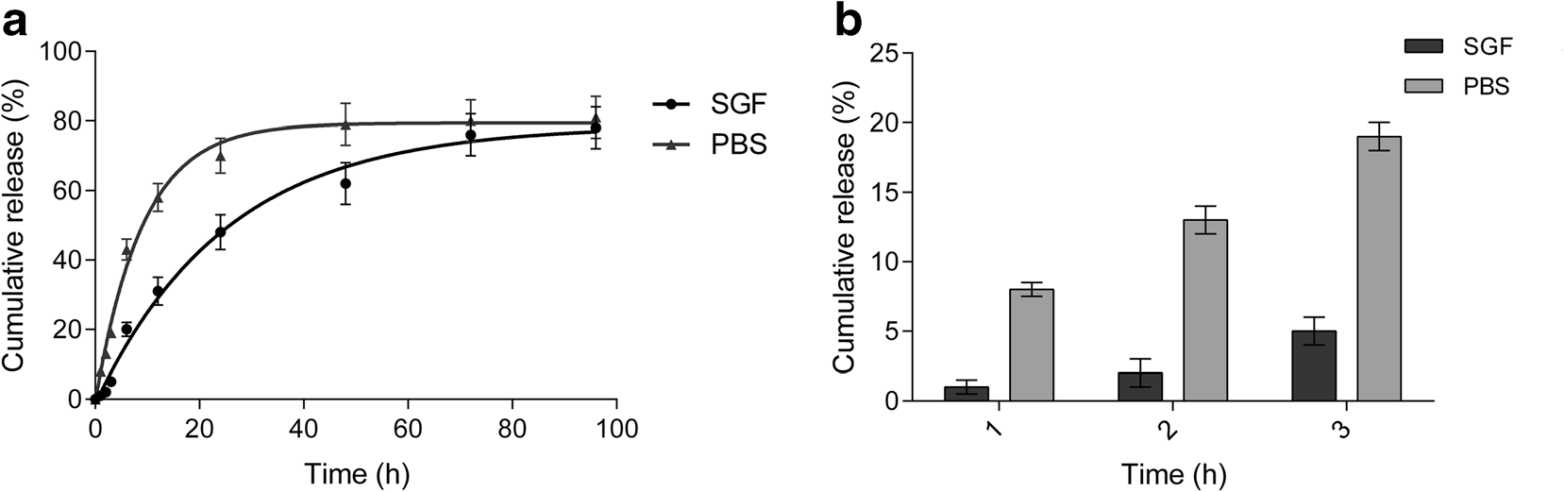
Release trend for TMZ from CS-SPLA nanoparticles in SGF (pH 2) and PBS (pH 7.4) at 37 °C. **a** Overall release; *R* ^2^ > 0.99, *k* = 0.009 in SGF, and *k* = 0.015 in PBS. **b** Amount of drug released in the first 3 h

The act of releasing TMZ from the CS-SPLA nanoparticles was performed in two different media. In order to evaluate the influence of pH, the authors made particular use of SGF and PBS. The cumulative release trend for the TMZ is shown in Fig. 4a, b. Both media had been prepared in accordance with European pharmacopeia standards.

As can be seen from the curves and *k* values obtained by Eq. 6, increasing the pH of the media results in the TMZ being released faster. In SGF, 50% of the loaded drug was released in almost 24 h, while this took place in PBS in less than 10 h. Moreover, as regards C_max_, 81% in PBS and 78% in SGF was reached in less than 2 days and after 3 days, respectively. As described in both our previous and other studies (Di Martino et al. 2016; Di Martino et al. 2015; Srivastava et al. 2016; Soares et al. 2016), such release is strictly bound with the swelling index; in polyelectrolytes, swelling is directly influenced by the pH and ionic strength of the surrounding environment. Furthermore, the presence of hydrophobic side chains affects the swelling ability and subsequently in the release of drugs, e.g., TMZ, which are mildly soluble in water (5 mg/mL).

In comparison to past studies on the release of anticancer drugs from polymeric nanoparticles (Win and Feng 2005), observation was made of reduced release intensity in the first 3 h after contact with the media, especially in SGF, where only 5% of the loaded drug was released after 3 h, whereas in PBS this totaled around 20%.

This finding indicates that most of the loaded TMZ is displaced in the inner section of the particles while there is only a small amount on the surface, the latter being immediately released after making contact with the media. The difference in intensity between PBS and SGF is related to the degree of protonation of the ionic groups along the CS backbone and CPLA side chains, which are responsible for electrostatic interactions of the intrachains and interchains. In acidic media, displacement of the media molecules through the polymer chains is delayed, or obstacles exist which reduce dissolution and cause subsequent diffusion of the TMZ molecules (Di Martino and Sedlarik 2014).

Delaying TMZ hydrolysis in MTIC (active metabolite) and AIC (inactive) is desirable, in particular under physiological conditions where pH >7 represents a challenge. The hydrolysis mechanism for TMZ has previously been described and thoroughly investigated through different methodologies (Denny et al. 1994; Wheelhouse and Stevens 1993; Lopes et al. 2013).

**Fig. 5:**
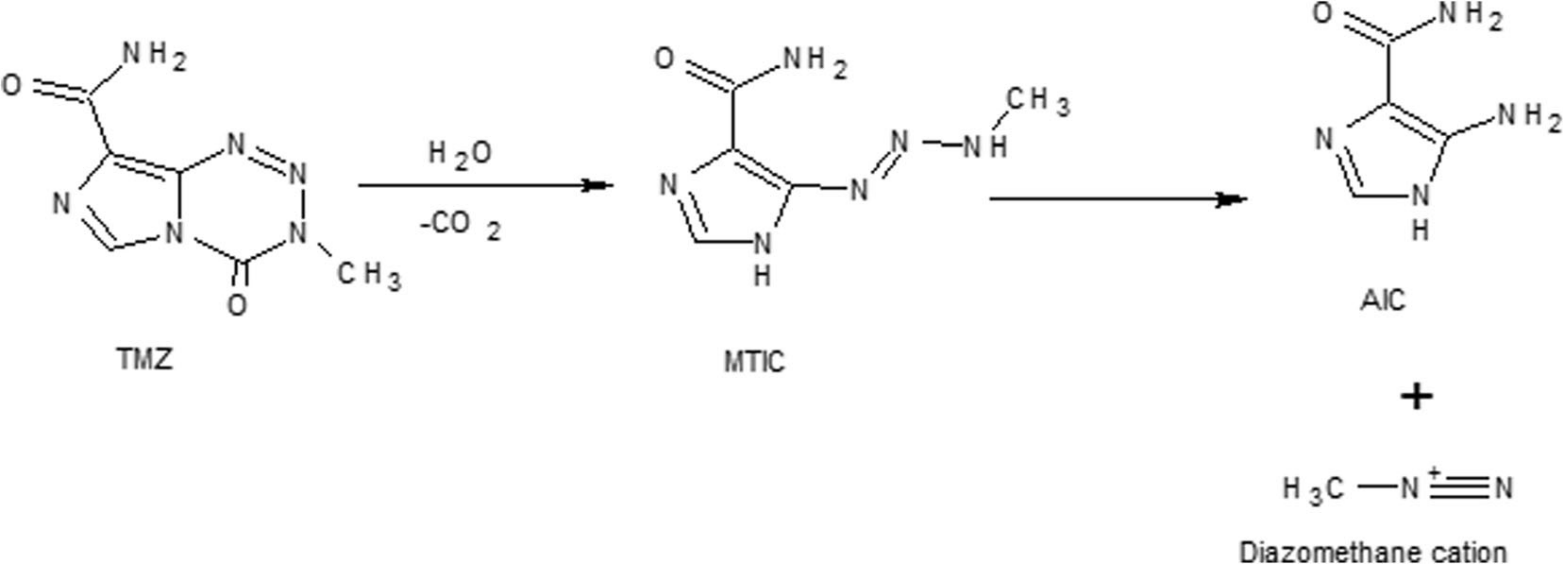
Schematic representation of TMZ hydrolysis

Figure 5 shows a simplified illustration of TMZ hydrolysis at pH >7.

**Fig. 6:**
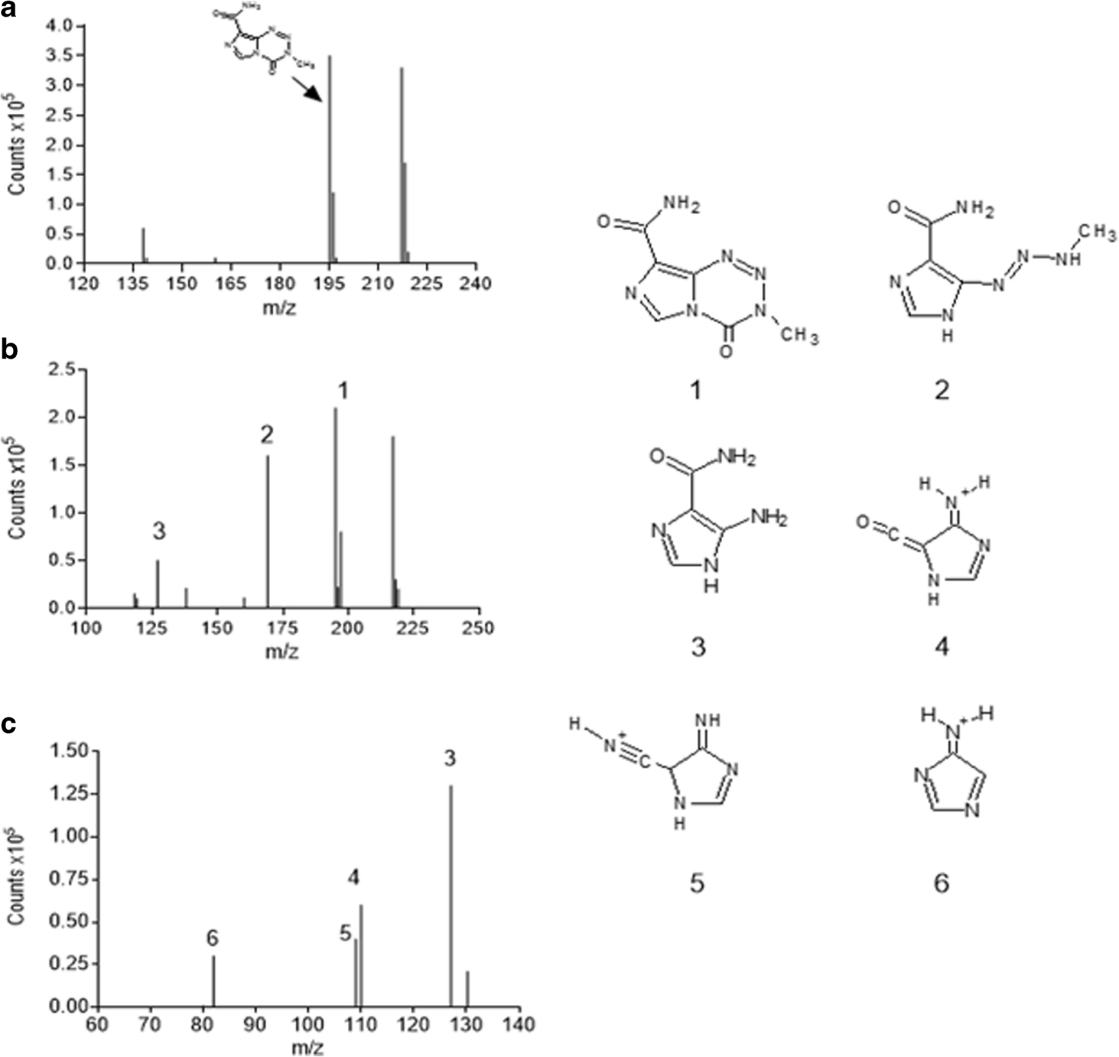
MS spectra for TMZ in aqueous solution at pH 7.4 at **a** time 0, **b** after 3 h, and **c** after 24 h. The reported chemical structures are *1* TMZ; *2* MTIC; *3* AIC; while *4*, *5*, *6* represent side products from MTIC degradation

TMZ ring-opening commences by adding a water molecule to carbonyl moiety. Afterward, intermediate (MTIC) is generated, resulting from elimination of CO_2_. MTIC decomposes quickly and releases methylated species (methyldiazonium ion) and 4-amino-5-imidazole-carboxamide (AIC); the latter is not active (Fig. 5). The mechanism of degradation is highly pH sensitive, the rate of TMZ increasing according to the pH of the media. The TMZ t_1/2_ in phosphate buffer of pH 7.4 stands at less than 2 h, while at pH < 4, it is up to 24 h (Kim et al. 2001). Moreover, other subproducts (Fig. 6) from MTIC degradation can be generated (Chowdhury et al. 1999).

In Fig. 6, the MS spectra of free TMZ over time in aqueous media at pH 7.4 is displayed.

Comparing the MS spectra in Fig. 6a–c, it is revealed that the degradation of TMZ in MTIC and AIC and other subproducts. At time zero (see Fig. 6a), in accordance with published works (Darkes et al. 2002; Di Martino et al. 2016), TMZ exhibits an ion at *m*/*z* 195 [M + H]^+^ and K adduct at 217, respectively. After 3 h of TMZ dissolution in the media, new peaks appear at *m/z* 169 (ascribable to [MTIC + H]^+^) and *m/z* 127, related to [AIC + H]^+^. However, TMZ still remains in the media. The presence of AIC is due to the low stability of MTIC, which tends to degrade in AIC over the period of minutes. After 24 h following dissolution (Fig. 6c), all the TMZ is hydrolyzed, and the main compound present in the media is highly stable AIC. The other peaks in Fig. 6c, in particular 4, 5, and 6, could relate to products resulting from the MTIC degradation of MTIC in AIC, as reported in previous studies (Jakobsen et al. 2001; Fiore et al. 1985).

**Fig. 7:**
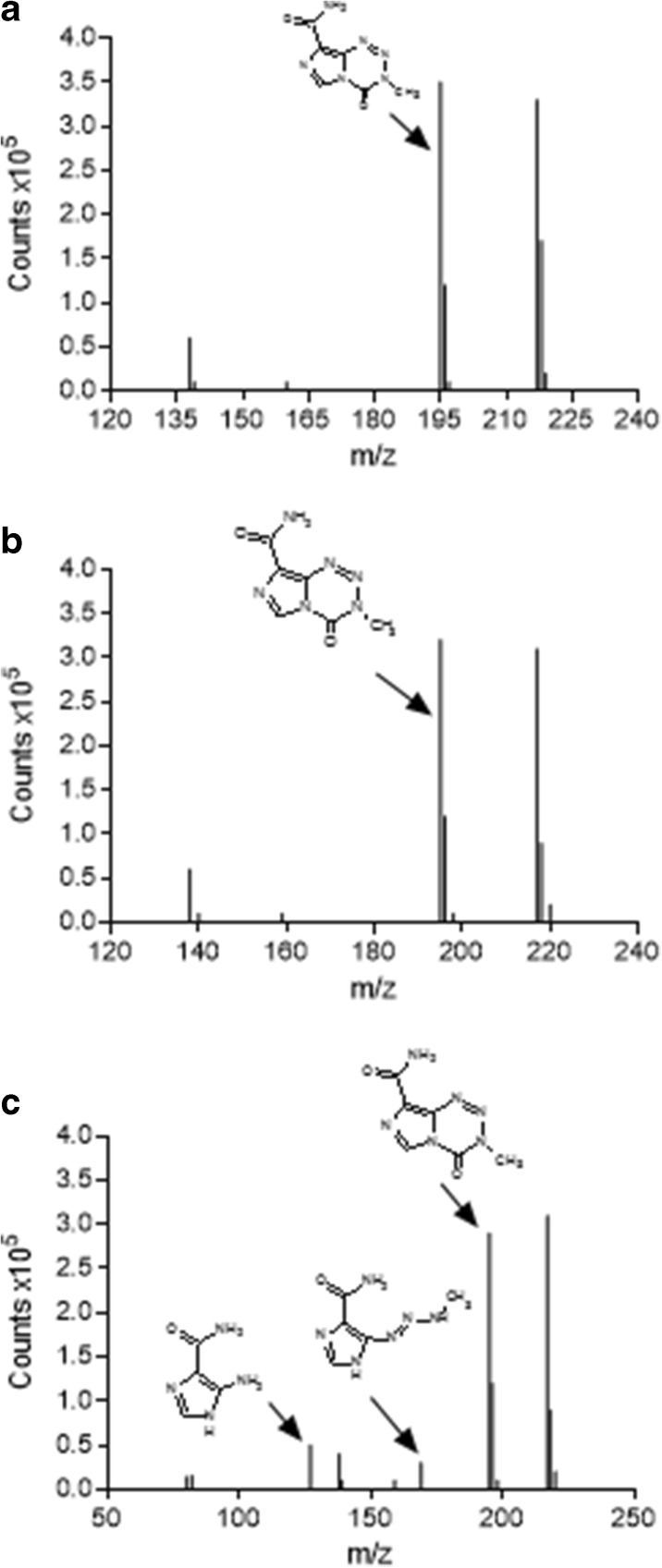
MS spectra for **a** TMZ as control and TMZ released from nanoparticles after **b** 6 and **c** 24 h in aqueous solution of pH 7.4

Figure 7 presents the MS spectra for the TMZ released from the CS-SPLA nanoparticles after 3 and 24 h.

A clear difference is visible between the spectra in Figs. 6 and 7. The latter reveals that the TMZ released after 6 h is still not hydrolyzed, indicating that the hydrolysis phenomena is considerably delayed when the drug is loaded in CS-SPLA nanoparticles. After 24 h (Fig. 7c), the peaks at *m/z* 195 (related to [TMZ + H]^+^) remain intense. However, the peaks referencing metabolites at *m/z* 169 [MTIC + H]^+^ and *m/z* 127 [AIC + H]^+^ are present.

**Fig. 8:**
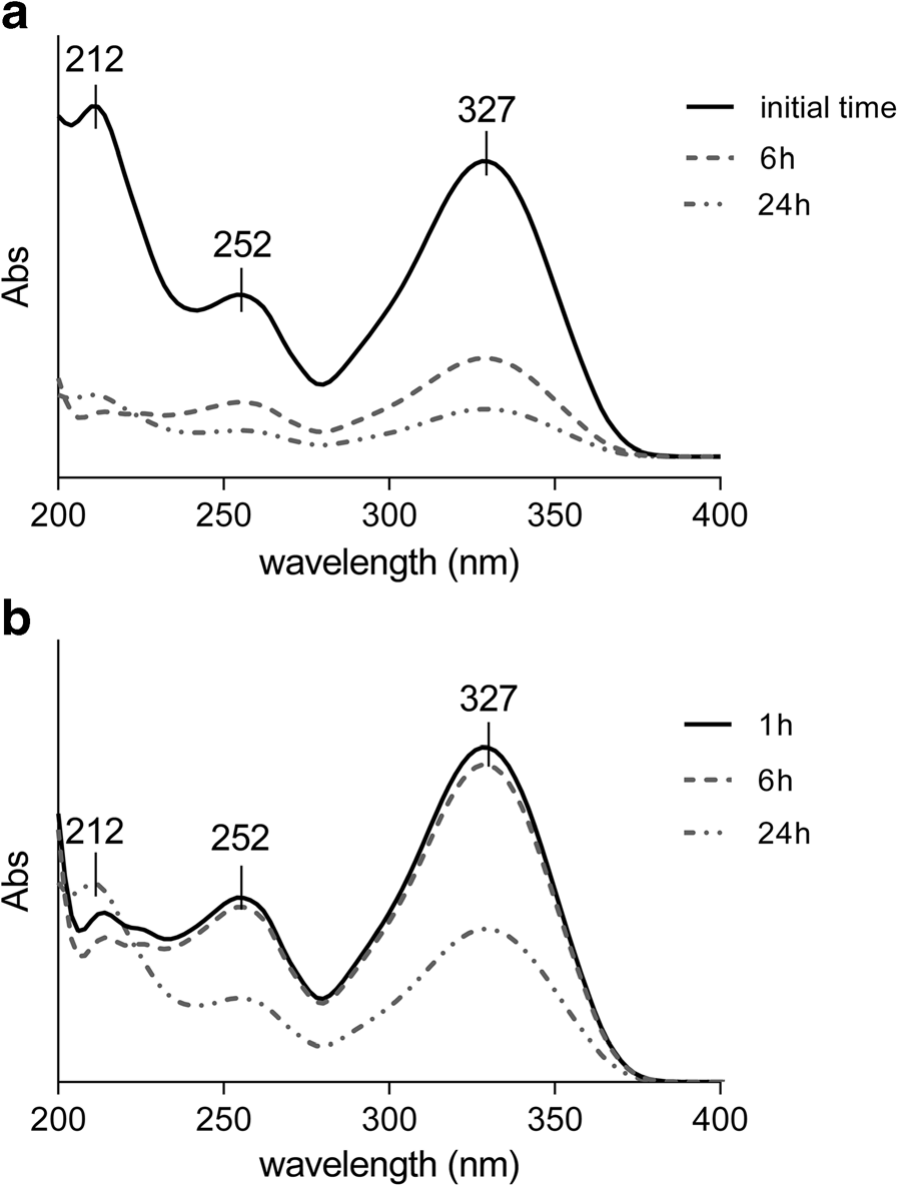
UV spectra for **a** free TMZ, control solution, and **b** TMZ released from nanoparticles. The pH level of the control solution and release media equals 7.4

These findings are also supported by UV analysis, as reported in Fig. 8.

Figure 8a displays the UV spectra for TMZ in pH 7.4 aqueous solution, collected at different times. Initially, the fresh solution presents three absorption bands at 212, 252, and 327 nm typical for TMZ, as reported in the literature (Lopes et al. 2013).

As can be seen, the intensity of the three bands decreases after 6 and 24 h, indicating hydrolysis of the TMZ, following the reaction illustrated in Fig. 5. Conversely, TMZ is preserved when loaded in the CS-SPLA nanoparticles, as after 6 h, no changes are observed in the intensity of band at the monitored wavelengths. Only after 24 h is a drop in intensity visible, in particular at 327 and 252 nm, indicating that hydrolysis has taken place. However, considering the MS results that confirm the structure of the TMZ and limited presence of hydrolysis products such as MTIC, AIC, or another subproduct, the diminished intensity of the band might relate to hydrolysis of TMZ release that occurred previously in the media, beyond the protection of the nanoparticles.

The MS and UV results reported herein demonstrate the delay of TMZ hydrolysis when loaded in CS-SPLA nanoparticles. Besides, several studies (Ananta et al. 2016; Mehare et al. 2015; Nygren and Eksborg 2012; Xu et al. 2016) dealing with encapsulation of TMZ in carrier to improve the therapeutical performances not so much is reported regarding the use of nano or micro carrier to reduce the hydrolysis of TMZ in its metabolites and prolong the half -life. The presented MS and UV results prove that by loading TMZ in CS-SPLA-based nanoparticles, it is possible to protect the prodrug from the external environment and delay the hydrolysis up to 24 h and preserving the therapeutic efficacy as reported in Fig. 10.

### Cytotoxicity and cell morphology evaluation

NIH/3T3 and MEF cell lines have been widely used for cytotoxicity testing for decades; cytotoxicity is evaluated as a decrease in cell viability (determined by an MTT assay) compared to the reference.

**Fig. 9:**
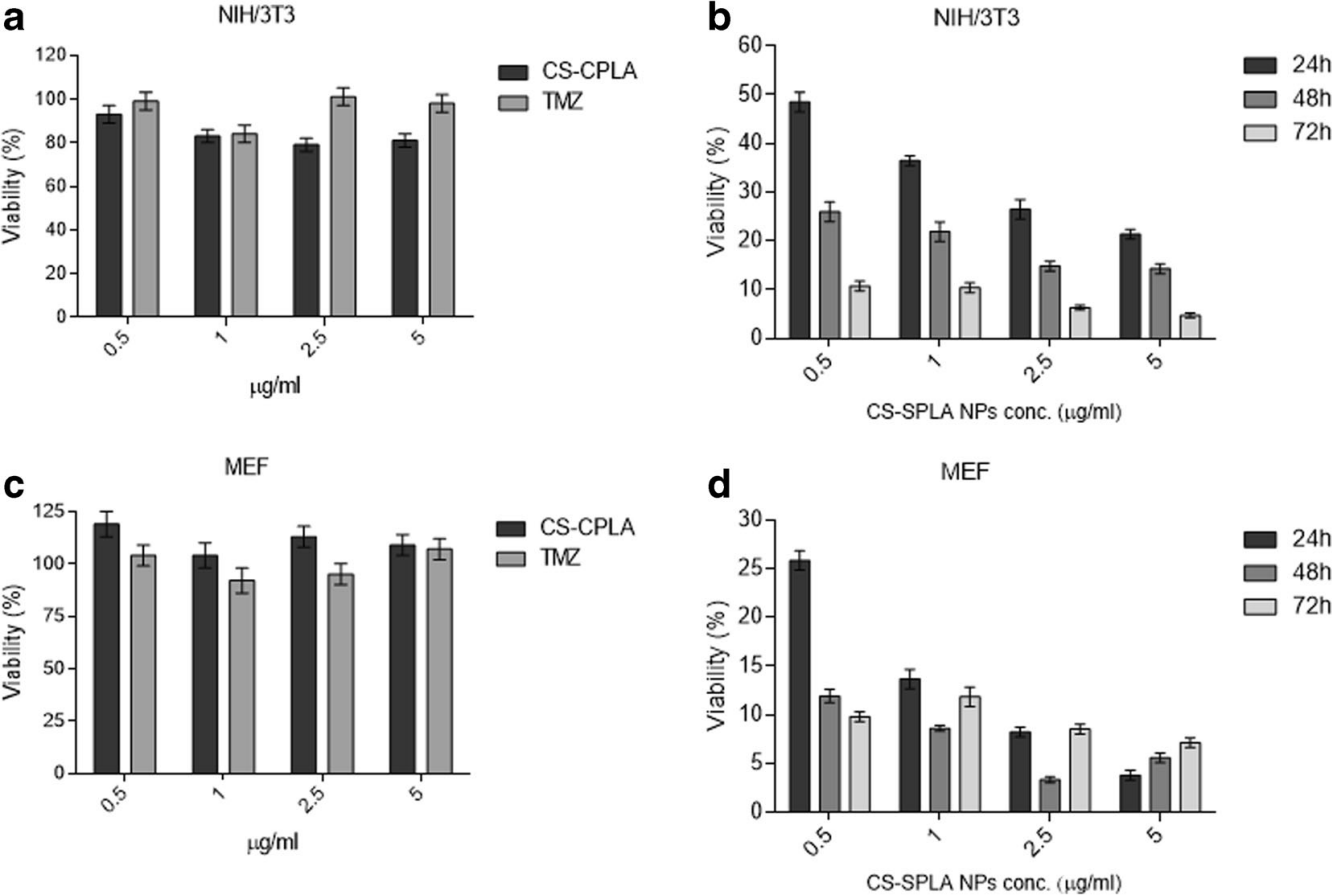
NIH/3T3 and MEF viability dependences on unloaded TMZ and bare CS-SPLA nanoparticles (**a**–**c**), and CS-SPLA nanoparticles loaded with TMZ, over time

The cytotoxicity studies (Fig. 9) showed the following: (i) TMZ exhibited no cytotoxicity when applied as the free drug, due to its hydrolysis into inactive products that occurs in less than 30 min after contact with physiological media at pH > 7; (ii) the viability of the cells was unaffected by the bare nanoparticles made of CS-SPLA in the range of concentration 0.5–5 μg mL^−1^ in the cultivation medium (viability above 80%).

As reported in Fig. 9, the viability of the NIT/3T3 and MEF cells dropped when TMZ was loaded into the CS-SPLA nanoparticles. Furthermore, cell viability tended to decrease over time, reaching values below 5% after 72 h. This clearly demonstrates that the prepared polymeric nanoparticles protect TMZ from hydrolysis. The significant impact of the nanoparticles in preserving TMZ from the hydrolysis is evident. Free TMZ quickly hydrolyse in the inactive metabolites in the time elapses between the sample preparation and the contact with the media, resulting incative. At the same time, when TMZ is loaded in CS-SPLA carrier, the hydrolysis does not take place preserving and prolonging the cytotoxic effect over time.

**Fig. 10:**
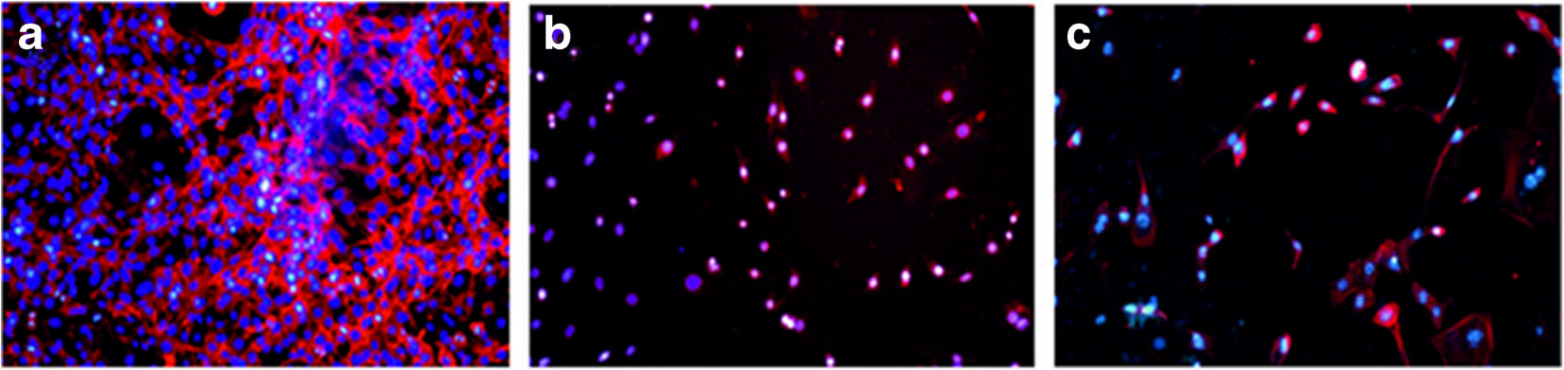
Inverted fluorescent microscopy observation of NIH/3T3 cells after **a** CS-SPLA, **b** CS-SPLA + TMZ (24 h), and **c** CS-SPLA +TMZ (48 h)

The capability of the carrier to preserve the structure and activity is additionally revealed by the micrographs (Fig. 10). Comparing the number of nuclei for pure CS-SPLA (Fig. 10a) with the carrier containing the drugs clearly shows that quantity of cells significantly diminishes (Fig. 10b, c). As the actin filamets are also counterstained, remarkable differences in cell growth, and spread are also obvious. Based on cell quantification as well as micrographs, it can be concluded that CS-SPLA-based nanoparticles demonstrably improve TMZ stability and preserving efficacy in terms of cytotoxicity is applicable in order to protect the structure of TMZ from hydrolysis and boost its cytotoxicity.

## Conclusions

The authors have described a novel kind of amphiphilic copolymer (CS-SPLA), which was obtained by grafting a natural polysaccharide CS with carboxy-enriched polylactic acid (SPLA) to load and improve the stability of the anticancer prodrug TMZ in physiological conditions. The resultant product maintains the properties of the main constituent, chitosan, in particular the cationic characteristics and the solubility. The average dimension and *ζ*-potential of the prepared carrier felt in the range 150–180 nm and 28–33 mV, respectively. TMZ was encapsulated with high efficiency, up to 80%, and no significant changes in the dimension and *ζ*-potential were detected after encapsulation. Moreover, the prepared system showed good stability in preparation media (pH 5.5) and physiological solution (pH 7.4) up to 1 month. MS and UV analysis clearly demonstrated the role of CS-SPLA in protecting and preventing hydrolysis of TMZ in MTIC, and subsequently in AIC in physiological conditions. It represents a great advantage as the main drawback of TMZ is the fast degradation in physiological conditions which causes a reduction in its therapeutic efficacy and an increase in the administration frequency. Release kinetics studies revealed the capability of regulating the release rate according to the pH of the external environment. The improvement in TMZ stability, but also the subsistence of its cytotoxicity effect when loaded into the CS-SPLA nanoparticles, was demonstrated by in vitro tests on NIH/3T3 and MEF cell cultures, where TMZ cytotoxicity lasted up to 72 h from the moment of contact. It gives a further confirmation of the ability of CS-SPLA nanoparticles to improve the hydrolytic stability of TMZ, which can enhance therapeutic efficiency of the cytostatic drug.

## Electronic supplementary material

ESM 1 (DOCX 116 kb)

## Abbreviations

^1^H–NMR: Proton nuclear magnetic resonance
AIC: 5-Aminoimidazole-4-carboxamide
CS: Chitosan
CS-SPLA: Chitosan grafted carboxy enriched polylactic acid
D: Molar mass dispersity
DLS: Dynamic light scattering
DNA: Deoxyribonucleic acid
DR: Drug released
DS: Dextran sulfate
EDC: 1-Ethyl-3-(3-dimethylaminopropyl)carbodiimide
EE: Encapsulation efficiency
FTIR-ATR: Fourier transform infrared-attenuated total reflection
GBM: Glioblastoma multiforme
GPC: Gel permeation chromatograpy
LC-MS: Liquid chromatograpy-mass spectrometry
MEF: Mouse embryonic fibroblasts
M_n_: Molar mass distribution
MTIC: 5-(3-Methyl-triazen-1-yl) imidazole-4-carboxamide
M_w_: Weight-average molecular weight
NHS: N-Hydroxysuccinimide
NIH/3 T3: 3-Day transfer standard fibroblast
PA: Pentetic acid
PBCA: Polybutyl cyanoacrylate
PBS: Phosphate buffer solution
PCS: Photon correlation spectroscopy
PLGA: Polylactic-co-glycolic acid
S: Swelling
SGF: Simulated gastric fluid
SPLA: Carboxy-enriched polylactic acid
TMZ: Temozolomide
UV-Vis: Ultraviolet-Visible spectroscopy
