# Supplementary material for: Enhancement of temozolomide stability by loading in chitosan-carboxylated polylactide-based nanoparticles
Source: J Nanopart Res. 2017 Feb 16;19(2):71. doi: 10.1007/s11051-017-3756-3 (PMC5313595; doi:10.1007/s11051-017-3756-3)
Supplement: Supplementary file 1 — (DOCX 116 kb) [file 11051_2017_3756_MOESM1_ESM.docx]

**^Supplementary material^**

**^Journal of Nanoparticles Research^**

**Enhancement of temozolomide stability by loading in chitosan-carboxylated polylactide-based nanoparticles**

Antonio Di Martino, Pavel Kucharczyk, Zdenka Capakova, Petr Humpolicek, Vladimir Sedlarik*

*Centre of Polymer Systems, University Institute, Tomas Bata University in Zlín, tr. T. Bati 5678, 76001 Zlin, Czech Republic*

**Figure S1 - ^1^H NMR spectra of neat linear PLA and functionalized non-linear PLA (SPLA).**

^1^H NMR measurements were performed on a Varian ^Unity^*Inova* 400 spectrometer. The chemical shifts of signals in spectra were referenced to solvent peaks (^1^H NMR (400 MHz, DMSO-*d*_6_)): δ=2.50 ppm). First order analysis was applied to evaluate all the NMR spectra received.


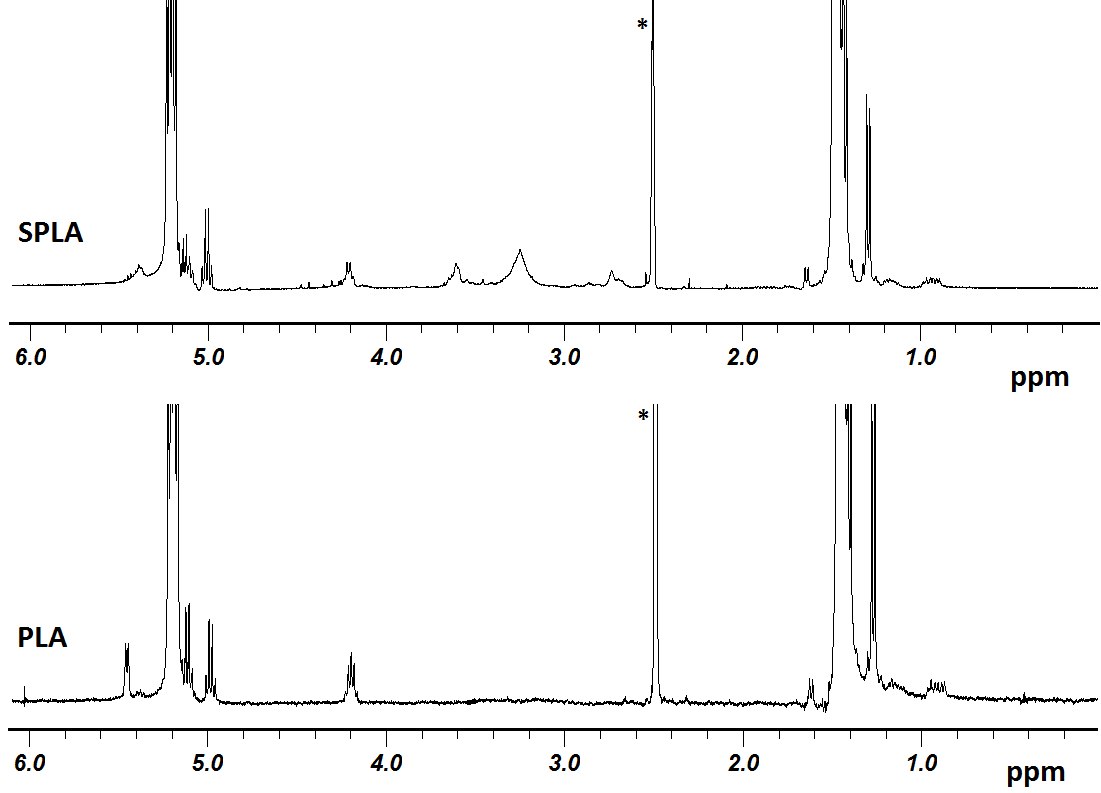


The measured ^1^H NMR spectra of linear and non-linear PLA are presented in Figure S1. As can be seen linear, PLA exhibits signals typical for this polymer; i.e. 5.18 ppm (CH) from the L-lactic acid repeat unit, 5.11 ppm (CH) from the D-lactic acid repeating unit, 5.0 ppm (CH) from the carboxylic acid chain end, 4.2 ppm (CH) from the hydroxyl-terminated chain end, 1.5-1.4 ppm (CH_3_) originating from the main chain, and 1.3-1.25 ppm for the (CH_3_) hydroxyl end and free lactic acid units. The detailed description can be found under Reference (*J.L. Espartero, I. Rashkov, S.M. Li, N. Manolova, M. Vert, Macromolecules 1996, 29, 3535*).

In case of SPLA two new relatively broad peaks appear around 3.22ppm and 3.6ppm. These are attributed to CH_2_ groups from pentetic acid (PA) and this can be taken as an qualitative evidence of presence of PA in molecular structure. After integration and of regions 5.0ppm ($A_{COOH}^{NMR}$ ) and 4.2ppm ($A_{OH}^{NMR}$) another information, which proof a non-linear character can be obtained. Because in linear PLA the number of COOH and OH end groups should be equal their ratio should be close to 1 (Table S1). In case of not linear structure with COOH enrich end group chemistry the ratio should be higher than 1, which was also detected by NMR (Table S1).

**Table S1. Properties of polycondensates calculated from ^1^H NMR spectra.**

|  | $\frac{{A_{COOH}^{NMR}}^{a}}{A_{OH}^{NMR}}$ |
| --- | --- |
| **PLA** | 0.98 |
| **SPLA** | 1.51 |

*a – molar ratio between carboxylic and hydroxylic end groups*

The signals of COOH and OH were derived from CH responses directly neighbouring with these terminal groups and are very easily detectable in case of low molecular weight PLA under normal conditions.
